# Medical Image and Physics Analysis of Human Physiology and Dysfunction

**DOI:** 10.12669/pjms.37.6-WIT.5005

**Published:** 2021

**Authors:** Shaofei Wu, Hailong Li

**Affiliations:** 1 Dr. Shaofei Wu Wuhan Institute of Technology, Wuhan, Hubei, China; 2 Dr. Hailong Li Cincinnati Children’s Hospital Medical Center, Cincinnati, USA

Human Physiology researches the life activity phenomena of the biological organism and the functions of the various components of the organism. The human body is mainly composed of organs with certain morphological characteristics and physiological functions, and any organ breaking away from the whole will cause physical dysfunction of the motor system, circulatory system, digestive system, respiratory system, urinary system, nervous system, endocrine system, and reproductive system, seriously affecting the normal work of the human body. Physiology studies the relationship between the human body and the environment at the overall level, the organ level, and the cellular and molecular level, the relationship between various systems in the body, the working mechanism of each system, the function of each sub-microstructure in the cell, and the special physical and chemical properties of each transformation of biomolecules. Traditional physiology mainly studies the law of life activities through various animal experiments, lacking direct observation of human life activities. In recent years, with the development of physics and engineering technology, experimental research on human physiology has not only clarified the principles of functional activities of various organ and tissue, but more importantly, it clarifies the basic law of life activities, and provide guidance for the study of other physiology topics. With the development of electronics, technologies such as telemetry and non-invasive body surface detection are becoming more mature, and the advancement of computer technology enables great progress in human physiology under various special conditions, and more in vivo physiological data can be obtained.

Medical images observe the internal tissue of the human body in a non-invasive manner. Traditional medical imaging results analysis relies on the doctor’s knowledge and practical experience, which is subjective with low efficiency. The increasingly high requirements on the analysis of medical clinical imaging results also promote the development of medical imaging. For example, in the process of multi-slice spiral CT scanning, isotropic voxel data can be collected in short time and reconstituted into three-dimensional data. A complete cardiac MRI examination results include hundreds of two-dimensional images at various moments, and on multiple levels. It is difficult to understand massive medical image-related information manually, and it increases the workload of doctors and seriously reduces work efficiency. With the development of computers and the emergence of digital instruments, analog images are converted into digital images for storage and transmission, which improves the accuracy and efficiency of disease diagnosis. As intelligent analysis and deep learning algorithms march forward continuously, medical image segmentation, image registration and information fusion, visualization, and functional analysis of time series images have been widely used in medical image processing, improving the quality of medical images and elevating the disease diagnostic efficiency. As computer technology marches forward continuously, medical imaging technology has become indispensable for clinical disease diagnosis, and medical image processing is essential in computer-aided diagnosis. Image segmentation refers to the automatic or semi-automatic extraction of the target area in the image to investigate the pathology and diagnose a certain disease. The medical images output by complex and advanced imaging instruments usually contain complex medical information, only relying on manual or semi-automatic segmentation will affect the accuracy, so the automatic segmentation of medical images is very important.

The medical imaging is no longer limited to diseases with obvious diagnostic characteristics in the past, but has begun to expand to a variety of different organs. Automatic and accurate quantitative computer-assisted image analysis elevates the accuracy, robustness, and efficiency of medical image analysis, laying a foundation for medical image quantitative analysis, three-dimensional visualization, and image-guided surgery. With the enrichment of image analysis and computer vision theoretical methods, there have emerged great breakthroughs in the segmentation of regions of interest in medical images. Medical image visualization technology can use medical image data obtained from experiments to reconstruct a three-dimensional image model for qualitative and quantitative analysis, which is convenient for doctors to observe and analyze from multiple angles and levels.

This special issue of Pakistan Journal of Medical Sciences focuses on human physiology and physiological dysfunction through medical imaging and physical analysis methods, covering medical functional imaging, medical image processing methods, medical image processing model establishment, computational fluid dynamics, new medical engineering materials, medical image signal processing, physiological signal monitoring, electrophysiology, and clinical physiology data analysis. The primary objective of this special issue is to outline the latest research progress in medical imaging, physics, and physiology, with special emphasis on new technologies to understand human physiology and new breakthroughs in research related to dysfunction in the past decade. It is a platform to display the work related to human physiology and physiological dysfunction. With the gradual transformation of the medical model to the bio-psycho-social medical model, more emphasis is placed on caring for patients, society, and the improvement of technology and services in diagnosis and treatment. People not only require minimally invasive or even non-invasive diagnosis and treatment methods, but also require early diagnosis and prevention of diseases. The help of medical imaging and physical analysis methods for clinical medicine is mainly manifested in the following aspects:

(1) A more reliable, detail and complete diagnosis and treatment basis can be obtained through medical imaging and physical analysis methods, so that the process of clinical diagnosis and treatment of diseases is non-invasive or minimally invasive acquisition of image information closer to pathology, effectively improving the level of diagnosis and treatment. For example, CTA has a huge advantage in the display of coronary arteries. This technology has unique functions for the display of calcification and myocardial bridge. CTA can detect soft plaques as small as 0.16 mm, which can guide timely interventional treatment. In addition, both MR and CT can perform myocardial perfusion imaging and cardiac function analysis, and obtain the perfusion status of myocardial capillaries and cardiac function parameters, etc., to provide more reliable imaging data and evidence for clinical treatment, thereby prompting treatment Indications and prognosis
(2) Medical imaging and physical analysis methods can detect and diagnose diseases early and improve survival rate. For patients with malignant tumors, the fundamental way to prolong survival lies in early diagnosis. For non-tumor diseases, such as acute cerebral thrombosis, myocardial infarction, pulmonary artery and peripheral vascular embolism, the effect of early thrombolysis is also very significant. Multi-layer CTA technology and MR diffusion-weighted imaging technology can make early diagnosis of vascular embolism diseases in time, and it is fast, simple and easy to perform. It can accurately locate diseased blood vessels and guide thrombolytic therapy.
(3) The continuous observation and follow-up of medical imaging and physical analysis methods have objectivity and reliability in the evaluation of curative effect, which can provide a basis for scientific and reasonable treatment and make the objective and accurate staging of malignant tumors a reality. According to the three-dimensional reconstruction technology in medical imaging and physical analysis, the tumor size changes during treatment can be accurately assessed. The development of functional and molecular imaging can even measure the degree of dispersion of water molecules inside the lesion before the morphological changes of the tumor, and then reflect it. Information such as the number of tumor tissue cells and changes in cell membrane integrity can also be used to measure tumor perfusion parameters, which indirectly reflect changes in micro vascular density and permeability, and can even assess changes in tumor metabolites during treatment. PET/CT imaging based on glucose metabolism has been widely used in clinical practice and has made a great contribution to the TNM staging of tumors.
(4) The medical imaging and physical analysis methods have resulted in important changes in the diagnosis and treatment model, such as minimally invasive medicine, multidisciplinary assistance, compound operating rooms, clinical section and imaging departments. Data transmission and sharing are becoming more and more convenient. Clinical diagnosis and treatment are increasingly dependent on imaging data. The information and diagnosis results provided by imaging can serve as a bridge to comprehensively analyze difficult cases from a systemic and overall perspective to guide the collaboration between multiple clinical disciplines. Medical imaging and physical analysis methods provide the basis for minimally invasive diagnosis and treatment, and minimally invasive techniques such as neural interventional, cardiac intervention, and peripheral vascular intervention have become the first choice for the treatment of corresponding vascular diseases.

This Special Issue will contribute a practical and comprehensive forum for exchanging novel research ideas or empirical practices of the medical image and physics analysis of human physiology and dysfunction.

The 31 original scientific papers included in this issue were carefully selected from a large number of manuscripts, after careful peer review process according to criteria of Pakistan Journal of Medical Sciences. We thank all the authors of papers, as well as the reviewers working on these articles. We also wanted to thank the Pakistan Journal of Medical Sciences, and personally its Chief Editor Mr. Shaukat Ali Jawaid for helping us to publish this issue.

